# Integrating Analytical Procedures in Routine Practices of Centralized Antiblastic Compounding Units for Valorization of Residual Compounded Drugs

**DOI:** 10.3390/pharmaceutics17010101

**Published:** 2025-01-14

**Authors:** Rita Patrizia Aquino, Giovanni Falcone, Paola Russo, Fabrizio Dal Piaz, Giulia Auriemma, Ferdinando Maria de Francesco, Stefania Cascone, Eduardo Nava, Pasquale Del Gaudio

**Affiliations:** 1 Department of Pharmacy, University of Salerno, 84084 Fisciano, Italy; aquinorp@unisa.it (R.P.A.); gifalcone@unisa.it (G.F.); gauriemma@unisa.it (G.A.); pdelgaudio@unisa.it (P.D.G.); 2 Department of Medicine, Surgery and Dentistry, University of Salerno, 84084 Fisciano, Italy; fdalpiaz@unisa.it; 3 Pharmaceutical Department, Local Health Authority Naples 3 South, 80059 Torre Del Greco, Italy; fm.defrancesco@aslnapoli3sud.it (F.M.d.F.); s.cascone@aslnapoli3sud.it (S.C.); e.nava@aslnapoli3sud.it (E.N.)

**Keywords:** personalized medicine, anticancer medicines, hospital compounded drugs, physicochemical stability investigation

## Abstract

Background/Objectives: Although extemporaneous formulations of anticancer drug products for personalized therapy are produced according to Good Hospital Pharmacy Manufacturing Practice, the lack of knowledge about drug stability under clinical conditions limits the second-time use of these highly costly medications in clinical practice. Therefore, the residual compounded drugs are considered waste and a cost item that negatively affects the healthcare system. In the context of the ever-increasing interest of the health system in applying practices in line with personalized medicine and spending review policies, this research aimed to demonstrate the feasibility of incorporating analytical techniques into daily routine practice. Specifically, the present research focused on fast stability analysis of Active Pharmaceutical Ingredients (APIs) in antiblastic residual compounded drugs with the purpose of demonstrating their potentialities as a resource for possible second-time use. Methods: Two different subsets of drug products were analyzed, i.e., medicines containing small molecules and medicines containing monoclonal antibodies. In relation to their different physicochemical properties, two analytical approaches were optimized and involved in the stability investigation: HPLC-DAD for small molecules and a combined approach of LC-MS/MS with size exclusion chromatography for monoclonal antibodies analysis. Results: Results underlined that the stability data, as available in the summary of product characteristics related to each medicine, do not completely describe the physicochemical shelf-life of anticancer compounded drugs. Conclusions: In fact, for all tested products, our results suggested a longer shelf-life in comparison to the datasheet, giving hospital pharmacists the possibility to extend the clinical use of compounded drugs, improving the cost–benefit of anticancer personalized therapy.

## 1. Introduction

The implementation of personalized medicine has the potential to improve patients’ quality of life and life expectancy while reducing financial and time costs [1]. Although personalized medicine offers significant potential for improving therapeutic outcomes, its integration into resource-limited healthcare systems poses significant challenges [2]. Optimizing the allocation of healthcare resources is essential to ensure effective and efficient service delivery [3]. Hospitals and other healthcare providers constantly work to ensure the best possible care for the patient while navigating a challenging business and economic landscape [4]. The challenges of balancing the quality and cost of patient care are even more relevant to the adoption of personalized medicine in medical subsets such as chemotherapy [5,6]. In fact, the ongoing development of targeted therapies and a multidisciplinary approach to new treatment modalities have contributed to improved outcomes for cancer patients [7,8], but also treatment costs (USD 30,790 of the eight-week treatment regimens providing Bevacizumab or Cetuximab compared to USD 63 for the same period of 5-fluorouracil, leucovorin, and oxaliplatin regimens). This trend underscores the need to adopt new policies aimed at improving cost-effectiveness, cost–utility, and cost–benefit [9,10]. In the context of personalized therapy, customized and prescription-based compounded drugs are prepared in hospital pharmacies, mixing individual ingredients in personalized doses and dosage forms to meet patient-specific needs. Therefore, a wide range of anticancer drugs need to be manipulated, i.e., reconstructed or diluted, by the hospital pharmacist before administration, transforming the raw medicines into extemporaneous pharmacy or magistral preparations. Compounding activity requires that regulatory aspects be considered, i.e., dose accuracy, sterility assurance, and stability under clinical conditions [11]. In many European countries, the reconstitution and preparation of anticancer drugs are carried out in centralized compounding units in a controlled and validated environment by expert personnel to ensure standardization. In fact, centralized manufacturing of anticancer medicine responds to specific regulatory and procedural guidelines that have been developed over the years in consultation with regulatory authorities. Centralized compounding is an effective strategy for ensuring product quality, workers’ and patients’ safety, and reducing the risk of environmental contamination while rationalizing pharmaceutical expenditure [12]. However, if dosage accuracy and sterility requirements can be considered guaranteed by the adoption of Good Hospital Pharmacy Manufacturing Practice (GHPMP) (GMP) for the quality assurance of the preparation in hospital pharmacies [13], with periodically verified procedures and operative personnel in the routine of centralized units, the stability of the compounded drugs under clinical conditions remains an issue that limits the possible second-time use of residual compounded drugs in clinical practice. In fact, depending on the prescribed dosage for specific patient clinical needs and the amount of Active Pharmaceutical Ingredient (API) available in the packaging of the medicinal product, after reconstitution and dilution, a certain fraction of the medicinal product persists from compounding activity [14]. However, the lack of stability data for compounded drugs forces pharmacists to consider residual compounded drugs as waste, which has a negative impact on the hospital’s bottom line. In fact, the stability data provided by the pharmaceutical industry in the summary of product characteristics (SmPCs) for each medicine are mostly related to the physicochemical stability of the raw drug product and the methods described in the pharmacopeias or ICH guidelines [15] do not fit the conditions of clinical practice [16]. The possibility of extending the validity of expensive medicine would be extremely helpful in providing hospital pharmacists with stability information on different drugs, as required for the proper management of a central compounding unit [17,18]. The availability of a longer period of use of the compounded drugs, which does not compromise the stability and safety of the drug, would facilitate the organization of centralized antiblastic units and would be economically advantageous, allowing the use of residual compounded drugs for subsequent preparations [19,20,21].

Considering this background, the present research, carried out in collaboration with the UFA Laboratory (Unità Farmaci Antiblastici-Centralized Antiblastic Drug Unit) of the Gragnano Hospital managed by the ASL Na3 Local Health Authority Naples 3 South, aimed to demonstrate the feasibility of incorporating analytical techniques into the daily routine practice of a hospital centralized compounding unit. The study focused on the optimization of rapid methods to assess the physicochemical stability of each compounded API in a 21-day investigation. Six anticancer medicines were used as models, i.e., ALIMTA^®^, ABRAXANE^®^, and JEVTANA^®^ as role models of small molecule drugs and HERCEPTIN^®^, AVASTIN^®^, and ERBITUX^®^ containing monoclonal antibodies. HPLC-DAD protocol was established for small molecule analysis, and a combined approach of LC-MS/MS with SEC (size exclusion chromatography) was used for monoclonal antibodies assessment.

## 2. Materials and Methods

### 2.1. Materials

All medicinal products were provided by the Health Authority Naples 3 South (ASL NA3Sud), Italy. According to their SmPCs, quali-quantitative and dilution data are listed below:

- ALIMTA^®^ 100 mg powder for concentrate for solution for infusion (Eli Lilly and Company, Indianapolis, IN, USA) (ALI). API: Pemetrexed disodium (Pemetrexed) at a final concentration of 25 mg/mL; other excipients: mannitol, hydrochloric acid, and sodium hydroxide [22];
- ABRAXANE^®^ (ABR) 5 mg/mL powder for dispersion for infusion (Bristol Myers Squibb, New York, NY, USA). API: Paclitaxel formulated as albumin-bound nanoparticles (Nab-Paclitaxel) at a final concentration of 5 mg/mL; other excipients: human albumin solution (containing sodium caprylate and N-acetyl-L-tryptophan) [23];
- JEVTANA^®^ (JEV) 60 mg concentrate and solvent for solution for infusion (Sanofi, Gentilly, France). API: Cabazitaxel at a final concentration of 10 mg/mL; other excipients: Polysorbate 80, citric acid, ethanol 96%, and water for injection [24];
- HERCEPTIN^®^ (HER) 150 mg powder for concentrate for solution for infusion (Roche Products Limited, Grenzach-Wyhlen, Germany). API: Trastuzumab, a humanized IgG1 monoclonal antibody produced by mammalian (Chinese hamster ovary) cell suspension culture and purified by affinity and ion exchange chromatography, including specific viral inactivation and removal procedures at a final concentration of 21 mg/mL; other excipients: recombinant human hyaluronidase (rHuPH20), L-histidine, L-histidine hydrochloride monohydrate, α,α-trehalose dihydrate, L-methionine, Polysorbate 20, and water for injections [25];
- AVASTIN^®^ (AVA) 25 mg/mL concentrate for solution for infusion (Genetech Inc., San Francisco, CA, USA). API: Bevacizumab, a recombinant humanized monoclonal antibody produced by DNA technology in Chinese hamster ovary cells, at a final concentration of 25 mg/mL; other excipients: trehalose dihydrate, sodium phosphate, Polysorbate 20, and water for injections [26];
- ERBITUX^®^ (ERBI) 5 mg/mL solution for infusion (Merck, Readington, NJ, USA). API: Cetuximab, chimeric monoclonal IgG1 antibody produced in a mammalian cell line (Sp2/0) by recombinant DNA technology, at a final concentration of 5 mg/mL; other excipients: sodium chloride, Glycine, Polysorbate 80, citric acid monohydrate, sodium hydroxide, and water for injections [27].

Solvents (acetonitrile, acetic acid, phosphate-buffered saline—PBS) and materials for chromatographic analyses (polyacrylamide gels, trypsin) were purchased from Sigma-Aldrich (St. Louis, MO, USA) and ultra-pure solvents for LC/MS analysis from Romil (Cambridge, UK).

### 2.2. Methods

#### 2.2.1. Residual Compounded Drugs: Shelf-Life Evaluation

All samples were prepared following the standardized protocols in use at the UFA Laboratory of the Gragnano Hospital:

ALI: The diluent, 4.2 mL of sodium chloride solution 9 mg/mL (0.9%) for injections, is injected into the Pemetrexed powder (100 mg) and gently stirred until complete powder dissolution with a final concentration of 25 mg/mL. The use of other solvents for reconstitution is avoided. The diluent is added slowly along the internal walls of the bottle, facilitating the dissolution of the powder with slow movements (at least 15 min) without shaking to avoid foam formation.

ABR: 20 mL of diluent solution is withdrawn with a syringe and injected through the spike very slowly into the API powder (100 mg of Nab-Paclitaxel) but along the internal walls of the bottle. The addition of the diluent is carried out in a time not shorter than 1 min. Once the syringe is disconnected from the spike, the latter is left inserted in the bottle for at least 7 min, slowly bringing it to a horizontal position and shaking. After the indicated time, the bottle is rotated with slow movements to avoid foaming, obtaining homogeneous dispersion (concentration 5 mg/mL) with a milky appearance and without visible precipitates.

JEV, the solvent, is quantitatively removed from the solvent bottle with a syringe and injected into the bottle containing Cabazitaxel (60 mg). Once the syringe and needle are removed, mixing is facilitated with slow, rotating movements of the vial without shaking to avoid foaming. Then, the solution (concentration of 10 mg/mL) is left to rest for at least 5 min until the solution appears clear, homogeneous, and with a light-yellow color.

HER: Trastuzumab (150 mg) was reconstituted with 7 mL of water for injections. The use of other solvents for reconstitution is avoided. This results in 10.2 mL of single-dose solution containing 21 mg/mL of Trastuzumab. The diluent is added slowly along the internal walls of the bottle, allowing powder dissolution with slow circular movements without shaking to avoid foam formation.

For AVA and ERB, no reconstitution is needed since the drugs are already in solution.

#### 2.2.2. Small Molecules Stability Assays

The chemical stability of each API (Cabazitaxel, Pemetrexed, and Paclitaxel) was performed by HPLC-DAD on Agilent Technologies HPLC, model LC 1220 Infinity, equipped with a DAD UV detector using an injection volume of 20 μL and setting the temperature at 25 °C. The main parameters of optimized methods are reported in Table 1.

For each API, the calibration curve in a range of concentration between 10 and 500 µg/mL was constructed, plotting peak area vs. concentration with six concentrations (10, 50, 100, 200, 300, and 500 μg/mL). Statistical analyses were performed using GraphPad Prism 10 software to evaluate the specificity, the calibration response, and the accuracy of optimized methods.

##### Dynamic Laser Scattering Analysis

The physical stability of Nab-Paclitaxel, formulated as albumin-bound nanoparticles, was investigated in terms of size distribution and surface potential of nanoparticles by Dynamic Laser Scattering using a Litesizer^TM^ 500 Particle analyzer (Anton Paar, Graz, Austria). Data acquisition was performed through Kalliope^TM^ software *v*.2.34.3. Analyses were conducted at 25 °C on Paclitaxel solution at 1 mg/mL (0.9 mg/mL of albumin) obtained by product dilution in 0.9% NaCl solution.

#### 2.2.3. Monoclonal Antibodies Stability Assays

The physicochemical stability of monoclonal antibodies was carried out by exploiting a combined assay: SEC and LC-MS/MS peptide mapping.

##### LC-MS/MS Peptide Mapping

**Figure 1:**
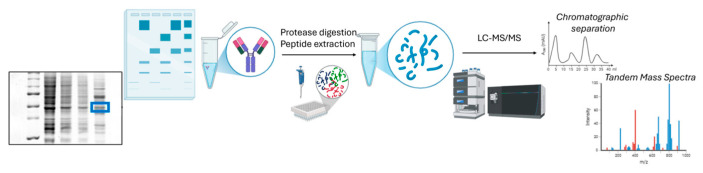
Protocol developed for protein peptide mapping.

Each monoclonal antibody was purified by one-dimensional electrophoresis on polyacrylamide gels. The band containing the proteins of interest underwent reduction and carboxyamidomethylation of cysteine residues and digestion using trypsin as a proteolytic enzyme, according to the method described in Figure 1.

The resulting peptide mixtures were analyzed using a system consisting of a nanoAquity (Waters, Milford, MA, USA) UPLC capillary chromatograph interfaced with a nanoSpray source mass spectrometer and equipped with an orbitrap-ion trap hybrid ion analyzer, Orbitrap XL (Thermo-Fisher, Carlsbad, CA, USA).

The data obtained were compared with those expected based on the amino acid sequence of the protein, thus revealing modifications of single amino acid residues or unexpected fragmentations of the polypeptide chain.

##### Size Exclusion Chromatography

For each monoclonal antibody, the hydrodynamic mobility, directly proportional to the conformation and the state of aggregation, was analyzed by molecular exclusion chromatography on a gel filtration column in Agilent Technologies HPLC, model LC 1220 Infinity. The column used was a Yarra3000 (300 × 4.6 mm; 3 µm), with a mobile phase consisting of phosphate-buffered saline (PBS) water solution and a flow rate of 0.5 mL/min. Before analysis, all samples were diluted from reconstructed concentration to a final concentration of 5 mg/mL, using an injection volume of 50 µL, and a spectrophotometric DAD detector settled at 280 nm to measure the absorption of the eluate.

#### 2.2.4. Twenty-One Days Physicochemical Stability

Residual compounded drugs, both from medicine containing small molecules and antibodies, reconstructed as reported in Section 2.2.1, were subjected to a three-week stability study (21 days). Three batches of each drug product were used in the present study. Taking into account instructions about storage conditions of each drug product reported in SmPCs [22,23,24,25,26,27], batches were stored in a clean area inside a pharmaceutical-grade refrigerator according to DIN 13277:2022-05 [28] at 4 °C protected from light, each one closed within a sterile, sealed bag. For all compounded drugs, by convention, the day of powder reconstitution will be defined as Day 1. Samples diluted to 1 mg/mL on Day 1 were the controls for the stability study. The drug stability analyses were performed on days 1, 2, 3, 5, 8, 10, 15, 18, and finally 21. Each analysis was carried out in triplicate, and data were reported as average value ± standard deviation. Considering the very narrow therapeutic range of chemotherapy agents and the risks associated with the degradation products (DPs), the limit of acceptability of tested products was set at 5% of the reference values (i.e., drug products on Day 1).

#### 2.2.5. Statistical Analysis

All statistical analyses were conducted using GraphPad Prism Software (v. 10.2). For methods validation, simple linear regression was carried out, choosing a confidence interval of 95% with a *p*-value of <0.05.

The one-way ANOVA (Analysis of Variance) test was applied to assess the significance of differences during stability studies (Tukey’s test α 0.05, *p*-value < 0.05).

## 3. Results and Discussion

The growing interest of healthcare providers in applying resource recovery policies underlines the need to identify methods that allow them to fill the gap between available data in the SmPCs of medicine and data about compounded drugs in clinical practices [29]. Compliance with good manufacturing practices for the quality assurance of the preparation in hospital pharmacies (GHPMPs [13]), notably in the reconstitution of pharmaceutical products, is a mandatory requirement aimed at ensuring safety in terms of dosage and microbiological steadiness. Therefore, the main issue that limited the implementation of the secondary use of these drug products was the absence of universal guidelines that ensure the quality of analysis to evaluate the shelf-life of antiblastic compounded drugs [14]. To overcome this limitation, starting in the past decade, many research groups worked to establish baseline unifying procedures applied in the physicochemical evaluation of antiblastic post-compounding formulations. Considering the high relevance of standard procedures, to carry out the present investigation, we followed “Methodological guidelines for stability studies of hospital pharmaceutical preparations Part 1: liquid preparations” disclosed by SFPC (French Society of Clinical Pharmacy) and GERPAC (Evaluation and Research Group on Protection in Controlled Atmosphere) [30].

The preliminary step of the present study was the selection of drug products that could be considered candidates for a resource recovery policy. Two different subsets of antiblastic drug products were evaluated, i.e., medicinal products containing small molecules or monoclonal antibodies, selecting three different medicines for each group. In both cases, medicines were selected according to the annual financial report of ASL Na3, in the function of the worst rating in terms of balance between frequency of use and costs. Products containing small molecules, Cabazitaxel, Pemetrexed disodium, and Paclitaxel (i.e., JEV, ALI, and ABR, respectively), are almost indicated as second-line treatment, thus representing expensive products in comparison to their frequency of use. On the other hand, considering the high costs of all products in the monoclonal antibodies subset, the selection of Trastuzumab, Bevacizumab, and Cetuximab in the related patented products was almost driven by the frequency of use.

Looking at the purpose of introducing analytic control into routine practices, the choice of specific analytical techniques is the result of a balance between the quality of the achievable results, the cost of running the instrumentation, and the ease of use. Thus, in the function of physicochemical differences between the two subsets, different analytical approaches were exploited to perform the stability study of the API in the residual compounded drugs: HPLC-DAD for small molecules and a combined approach of LC-MS/MS with SEC for monoclonal antibodies. Thus, results obtained from each drug product subset will be discussed separately.

### 3.1. Compounded Drug Products Containing Small Molecules

Usually, in a stability study performed for research purposes, the selection of the technique is driven by the physicochemical properties of the compounds to be determined. However, as also described in GERPAC guidelines, in hospital pharmacies, this selection is additionally dictated by what equipment is available and the costs connected to its routine use. For these reasons, HPLC is more recommended than other techniques, allowing the analysis of a very broad range of small molecules with different physicochemical properties and restrained running costs [30].

Starting from these premises, an HPLC-DAD method was selected and fine-tuned to provide an accurate response in a timely manner that fits with the practical needs of the hospital activities, performing serial analyses on several products using similar equipment and conditions (mobile phase, column system, and detector), in a short time.

Each drug product ALI, ABR, and JEV immediately after reconstruction was diluted to obtain a concentration of 1 mg/mL and used as a reference.

#### 3.1.1. Methodological Approach to Define Small Molecules Chemical Stability Assays

In the U.S. Pharmacopeia 44th edition, there are specific monographies about Pemetrexed disodium and Paclitaxel; however, no info is available in the compendia for Cabazitaxel.

According to the USP method of Pemetrexed disodium [31], the following apparatus was used: C-8 reversed-phase column, DAD detector settled at 285 nm, mixture of acetonitrile and water buffer (i.e., water added with acetic acid 0.1% *v*/*v* to obtain a pH around 5.3) as mobile phase.

In detail, different ratios of the solvent mixture were evaluated from 1.0:9.0 to 2.5:7.5, varying also the flow rate from 0.8 to 1.2 mL/min, aiming to obtain API elution time of less than 10 min. The best results were obtained using a mobile phase in isocratic elution, consisting of 1.3 parts acetonitrile and 8.7 parts buffer, and a flow rate of 1.0 mL/min. In these conditions, the complete elution of the active ingredient from the column occurred in about 7 min.

A similar approach was exploited to find the optimal method set-up for the evaluation of Paclitaxel. According to the USP [31], the HPLC system was equipped with a C-18 reversed-phase column, and the DAD detector was settled at 228 nm. The mobile phase was an acetonitrile/water mixture in a ratio of 6.0:4.0. Testing different flow rates in a range between 1.0 and 1.5 mL/min and setting the final flow rate at 1.5 mL/min, a retention time of 10 min was obtained.

Differently from Pemetrexed and Paclitaxel, no information on quantitative assays of Cabazitaxel in official compendia is available, so two specific studies were considered the basement of the present study [32,33]. As a consequence, the following instrument configuration was settled: C-18 reversed-phase column, DAD detector (232 nm). Different mixtures of acetonitrile/water at a ratio between 9.0:1.0 and 1:1 *v*/*v* and a flow between 1.0 and 1.5 mL/min were evaluated, establishing as an optimal condition a mixture of 8.0:2.0 at 1.5 mL/min that allows us to obtain a very short retention time around 4 min.

The selectivity of each method was evaluated by the comparison of API and mobile phase chromatograms: no peak was detected at the retention time of each small molecule in the chromatograms of mobile phase blank. The calibration response was evaluated through linearity regression in a range of concentration from 10 to 500 μg/mL. Each linearity regression coefficient is listed as follows: Pemetrexed r^2^ = 0.9975, Nab-Paclitaxel r^2^ = 0.9998, and Cabazitaxel r^2^ = 0.9991. Finally, the accuracy of each optimized method was evaluated by comparing the observed concentration with the theoretical concentration for all points of a calibration curve. In all cases, the statistical analysis highlighted non-significant differences between theoretical and observed concentrations (*p* < 0.05).

#### 3.1.2. Chemical Stability Investigation of Compounded Drug Products Containing Small Molecules

The developed methods were used to analyze the chemical stability of each API in hospital drug residues daily compounded over three weeks. The evaluation was carried out starting from Day 1. Samples were diluted at a final concentration of 200 μg/mL, then several aliquots were picked up, and each one was analyzed at a different time (Table 2).

Results obtained from the stability studies of ALI shown in Table 2 evidenced a non-significant reduction in the Pemetrexed content up to 5 days of storage, less than 0.5% (99.72% recovery in API). After 5 days, a continuous reduction in drug content was observed: more than 1% after 8 days until it reached the 5% limit on the 21st day. SmPC of ALIMTA^®^ reports the shelf-life of the reconstituted compounded drug as 24 h at refrigerated temperature (SmPC, Section 6.3 [22]), but our results highlighted that the chemical stability of Pemetrexed was ensured until three weeks after reconstitution if stored correctly at 4 °C.

Moving the attention to Nab-Paclitaxel (ABR), the HPLC-DAD investigation revealed a non-significant variation in API content up to 3 days of storage, followed by a minimum decrease on Day 4. On the other hand, from Day 5, it is possible to observe a relevant drop in the drug concentration from 5% in one day to a 35% drop after Day 15. To better highlight the mechanism behind this rapid down-drop of Nab-Paclitaxel concentration after Day 4, a Dynamic Laser Scattering study was performed to evaluate the physical stability. Nab-Paclitaxel particles on Day 1 showed a mean diameter of about 130 nm with a narrow size distribution (PDI < 0.1), while an apparent zeta potential of a −23 ± 3.1 mV was detected. This observation is in line with the presence of a partial negative charge on the particle surface due to interactions between the Paclitaxel and the albumin matrix in the nanoparticle structure. On Day 4, changes in both particle size and zeta potential were observed: particle size increased up to 85% until 220 nm diameter, PDI > 0.4, and the zeta potential moved to −22 ± 2.4 mV. Zeta potential reduced its absolute value over time until reaching a minimum value of −0.8 ± 2.4 mV after 15 days, suggesting a partial degradation of the Paclitaxel chains linked to the surface of the albumin nanoparticles. Considering that the shelf-life of ABR after the opening of reconstituted dispersion in the vial or of the infusion bag is 24 h at 2–8 °C in the original container and 24 h at 2–8 °C followed by 4 h at 25 °C, protected from light (as reported in Section 6.3 of its SmPC), the shelf-life of residual compounded drug comprehensive of nanoparticles stability until 3 days is still a very promising result.

Finally, the results of the Cabazitaxel (JEV) investigation indicated a very limited reduction in the API content over the first 8 days. Indeed, as shown in Table 2, Cabazitaxel content remains unchanged after 24 h of storage at 4 °C, and after 8 days of storage, it decreased by only 1.1%. Interestingly, the reduction in Cabazitaxel content has an almost linear relationship with time up to Day 15 (97.58% content). Although the in-use shelf-life of JEVTANA^®^ after dilution is reported to be 1 h at ambient temperature and 24 h at 2–8 °C (as reported in Section 6.3 of its SmPC), our results about the chemical stability of Cabazitaxel open the possibility of using a residual compounded drug for a very long time (until 18 days); however, further studies are needed in order to make a conclusion.

### 3.2. Compounded Drug Products Containing Monoclonal Antibodies

In comparison to small molecules, protein drugs can undergo more complex degradation pathways related to both physical and/or chemical stress [34]. Moreover, the ability of a protein API, such as monoclonal antibodies, used in the therapeutic field to interact with its targets and partners is strictly associated with both the primary (amino acid sequence and any post-transcriptional modifications) and tertiary structures (conformation and intermolecular interactions). To evaluate whether the stability of these protein APIs is preserved even after any stress, it is necessary to verify that primary and tertiary structures are still retained [35]. In recent years, some research groups focused their attention on the development of methods to evaluate the stability of monoclonal antibody products after reconstruction [36]. For example, Vieillard et al. evaluated the stability of Cetuximab in opened vials exploiting a multiple techniques approach, i.e., SEC, DLS, derivative UV spectrophotometry, and fluorescence spectroscopy [37]. Carpanese et al. proposed an orthogonal assessment of the physicochemical, biological, and microbiological properties of ERBITUX^®^ and VECTIBIX^®^ in original opened glass vials [38]. They analyzed the tertiary structure changes in UV spectroscopy between 250 and 320 nm in second derivative mode, while changes in primary structure were evaluated by combining SDS_PAGE and SEC-HPLC. In both cases, an accurate evaluation of monoclonal antibody stability was performed. However, considering the final aim of the possible introduction of these investigations in daily routine practices of anticancer centralized units, the analytical techniques involved could not fit the daily routine. Thus, in accordance with the guidelines by GERPAC that suggested the possibility of using different analytical techniques as instruments for stability investigation, SEC and LC-MS/MS combined approaches were applied. In detail, SEC was exploited to evaluate the tertiary structure stability of monoclonal antibodies, while LC-MS/MS was used as a detection technique in a peptide mapping protocol settled to evaluate the stability of the primary structure. LC-MS/MS could be easier to introduce in routine practice for stability studies due to the high resolution of the process, as well as the shorter analysis times compared to ionic exchange chromatography, which is defined as the golden standard by GERPAC.

The studies carried out in this phase were conducted on residual compounded drugs Trastuzumab (HER), Bevacizumab (AVA), and Cetuximab (ERB).

#### 3.2.1. Methodological Approach to Define Monoclonal Antibodies Stability Assays

To perform mAbs primary structure stability evaluation, it was necessary to optimize the peptide mapping approach. As well known, prolonged storage of proteins in aqueous solutions can also cause changes in their chemical structure; oxidation of methionine residues, deamination of glutamine residues, and hydrolysis of some peptide bonds (primarily those between asparagine and proline residues) are the most common events. The most effective analytical approach for the analysis and verification of the primary structure of a protein is the accurate measurement of its molecular weight coupled with the so-called peptide mapping. This technique is based on enzymatic digestion of the protein, aimed at generating peptide fragments that cover the entire sequence of the protein itself, and on their subsequent analysis using liquid chromatography techniques coupled with LC-MS/MS tandem mass spectrometry [39].

The method developed (Figure 1) involved the purification of each protein to be analyzed by one-dimensional electrophoresis on polyacrylamide gels, reduction and carboxyamidomethylation of cysteine residues, and digestion in gel, using trypsin as a proteolytic enzyme. The peptide mixtures generated were analyzed by a UPLC capillary chromatograph interfaced with a nanoSpray source mass spectrometer and equipped with an orbitrap-ion trap hybrid ion analyzer [40,41]. The comparison between the obtained data and those expected based on the amino acid sequence of the protein allowed us to highlight possible modifications of single amino acid residues or possible fragmentations of the polypeptide chains.

Preliminarily, full MS spectra of the three proteins were acquired. MS spectrum obtained from the Trastuzumab analysis showed the presence of a main species with a molecular weight—calculated from the average of three measurements—of 145,534 ± 4 g/mol (Trastuzumab theoretical weight 145,532 g/mol). The Bevacizumab spectrum showed a species with a molecular weight of 149,195 ± 4 g/mol (theoretical weight Bevacizumab 149,197 g/mol) and the Cetuximab a species with a molecular weight of 145,783 ± 5 g/mol (theoretical weight Cetuximab 145,782 g/mol). Subsequently, in-gel digestion coupled with a mass spectrometry approach was used to analyze samples containing Trastuzumab, Bevacizumab, or Cetuximab. The data obtained, processed manually and with the support of the Paws software, allowed us to confirm that this method was suitable to verify the amino acid sequence of the three proteins (Table 3, Table 4 and Table 5).

Results demonstrate that the obtained data allows the verification of the correctness of more than 80% of the amino acid sequence of the various proteins and is therefore suitable for the evaluation of the chemical stability of monoclonal antibodies and other proteins for therapeutic use.

Moving on to the investigation of tertiary protein structure, stability was investigated via SEC, applying an indirect quantization method. In detail, gel filtration chromatography equipped with UV-DAD detection was exploited to evaluate the hydrodynamic mobility, which was directly proportional to the conformation and the state of aggregation of the analyzed proteins. To optimize and verify this approach, for each mAbs, the exact retention time and the profile were determined on Day 1 and after 10 days of exposure to samples at stress conditions, i.e., light, not controlled humidity, and room temperature. Comparing obtained chromatograms, it was possible to observe no changes in both retention time and peak profile clearly attributable to the formation of oligomers of the analyzed monoclonal antibodies, confirming the accuracy of the present method to detect any changes to the tertiary structure.

#### 3.2.2. Stability Investigation of Compounded Drug Products Containing Antibodies

We used the developed method to analyze the residual compounded HER, AVA, and ERB from compounding in Gragnano Hospital stored at 4 °C for three weeks.

For HER, the chemical and physical stability of the reconstituted solutions in water for injection under aseptic conditions is reported in its SmPC to be 48 h at 2–8 °C. Interestingly, after aseptic dilution in polyvinylchloride, polyethylene, or polypropylene bags containing sodium chloride 9 mg/mL (0.9%) solution for injection, physicochemical stability has been demonstrated at 24 h at temperatures not exceeding 30 °C and up to 30 days at 2–8 °C [17,42].

Regarding AVA, available data in its SMPC on physicochemical in-use stability of the diluted medicinal product is reported as 30 days at 2 °C to 8 °C plus an additional 48 h at 2 °C to 30 °C in sodium chloride 9 mg/mL (0.9%) solution for injection.

Considering the chemical and physical in-use stability of ERB, the expiration date is after 48 h at 25 °C.

Peptide mapping investigation of residual compounded drugs highlighted no changes in the primary sequence of the antibodies among data obtained during the time frame of the present stability study. In detail, LC-MS/MS analyses revealed that in all three cases, the average molecular weight did not vary significantly over the period analyzed (21 days, Table 6), demonstrating that no modification of the primary structure of the proteins occurred.

Finally, results obtained from gel filtration analysis demonstrated that for none of the three antibodies, it was possible to observe significant variations in the state of oligomerization during the entire investigation period. Retention time, intensity, and shape of the chromatographic peaks relating to each species do not show significant changes, suggesting the conformational stability of the antibodies during the three weeks of storage at 4 °C. This finding is very interesting considering the short half-life normally defined for compounded drug products containing antibodies, though depending on the product.

We have to consider that the short half-life indicated for this subset of antiblastic medicine is largely based on the possible biological risk and not on intrinsic physicochemical instability. However, results from the present research suggest that the selected monoclonal antibody-based compounded drugs have stability over time greater than that reported, and the residues can be safely used, providing that preservation in aseptic conditions can be guaranteed.

## 4. Conclusions

The ever-increasing adoption of personalized medicine approaches underlines the need for healthcare providers to optimize the balance between the quality of patient care and the financial aspects of the healthcare system. Resource recovery policy could be a valid pathway to meet this aim.

This policy became more relevant in application to the extemporaneous magistral formulation of antiblastic medicines characterized by high costs, high frequency of administration to different patients in different doses, and large waste production. In fact, the stability of antiblastic compounded drugs, frequently established to a few hours/days for microbiological needs, becomes a limiting factor to clinical practice, whereas the physicochemical stability of specific molecules may be much longer, and the content of API may remain steady in compounded formulations. However, the limit for a second use is still in the absence of universal guidelines that describe procedures to be applied for the evaluation of the stability of drug product residues, and, as a consequence, residual compounded drugs are considered waste.

The actual shelf-life of compounded drugs must be evaluated by analytical techniques, i.e., HPLC-DAD, or LC-MS/MS, with good sensitivity and capability of identification of target analyte and effective in protein analysis (LC–MS/MS). These techniques can be integrated into the routine practices of centralized compounding units to perform fast physicochemical stability assays of daily residual compounded drugs.

In this respect, results reported in the present paper, though at a preliminary stage, are very informative for the daily practice of hospitals and may offer a tool for the safe and efficient reduction of waste production and disposal and re-use of the preparations because the drug sensitivity to physicochemical factors during the preparation, managing, and storage phases seems to be limited for the studied API.

In fact, the present case study showed how several compounded drugs with different physicochemical characteristics are stable until three weeks post-compounding.

The re-use for other patients represents a potentially valuable resource to enhance cost-effectiveness in personalized anticancer therapy.

In particular, considering the possible shelf-life extension overall evidenced by this case study in relation to the annual financial report of ASL Na3, comprising medicines frequency of use and costs, it is possible to forecast an annual economic resource recovery for these medicines of about EUR 820,000, taking into account the total value recovered for potential medicine packages saved and the total value recovered for potential residual product secondary use. At this point, an optimization of the workflow and working time must be added, as well as a reduction in the costly operators’ engagement.

These interesting results obtained integrating a centralization of drug administration with a proper re-use of residuals can be a valid foothold to the hospital pharmacist for a conscious decision to go beyond the expiration date reported in the SmPCs of a specific anticancer medicine and to obtain economically advantageous exploiting a potential extended use of the residues.

Other studies focusing on the clinical outcomes are necessary for an extended adoption in clinical practice in the near future, hoping for concrete support from regulatory agencies in the definition of standard guidelines.

## Figures and Tables

**Table 1:** Optimized HPLC-DAD set-up for each small molecule: Pemetrexed, Paclitaxel, and Cabazitaxel.

	Pemetrexed	Nab-Paclitaxel	Cabazitaxel
Chromatographic column	Phenomenex Luna C8 4.6 × 250 mm, (5 µm)	Agilent SB C18 4.6 × 250 mm, (5 μm)	Agilent Zorbax Eclipse XDB-C18, 4.6 × 250 mm, (5 μm)
Mobile phase composition	Acetonitrile/Buffer ^1^ (1.3:8.7)	Acetonitrile/Water (4.0:6.0)	Acetonitrile/Water (8.0:2.0)
Flow rate (mL/min)	1.00	1.5	1.5
DAD detector wavelength (nm)	285	228	232

^1^ Buffer was water added with acetic acid 0.1% *v*/*v*.

**Table 2:** Chemical stability data over 21 days of small molecules containing products: Cabazitaxel, Pemetrexed disodium, and Nab-Paclitaxel by HPLC analysis.

	Pemetrexed (% ± st. dev.)	Nab-Paclitaxel (% ± st. dev.)	Cabazitaxel (% ± st. dev.)
Day 1	100.00 ± 0.09	100 ± 0.31	100.00 ± 0.01
Day 2	100.00 ± 0.11	99.93 ± 0.47	100.00 ± 0.01
Day 3	99.97 ± 0.22	99.92 ± 0.53	99.80 ± 0.02
Day 4	99.72 ± 0.32	99.79 ± 0.92	99.22 ± 0.02
Day 5	99.34 ± 0.41	93.75 ± 1.02	99.10 ± 0.01
Day 8	98.94 ± 0.48	92.67 ± 2.51	98.90 ± 0.02
Day 10	97.96 ± 0.51	86.97 ± 2.55	97.95 ± 0.03
Day 12	97.70 ± 0.52	80.12 ± 2.75	97.50 ± 0.03
Day 15	97.58 ± 0.58	65.76 ± 3.53	97.20 ± 0.04
Day 18	95.00 ± 0.62	-	96.80 ± 0.03
Day 22	94.78 ± 0.63	-	94.60 ± 0.03

**Table 3:** Peptides identified after digestion of Trastuzumab with trypsin by LC/MS/MS analysis.

Light Chain		Heavy Chain	
Measured Molecular Weight (Da)	Peptide Length	Measured Molecular Weight (Da)	Peptide Length
1877.9	1–18	1881.1	1–19
691.4	19–24	1109.6	20–30
1989.5	25–42	1088.6	31–38
1771.9	46–61	829.5	44–50
4129.9	67–103	1083.5	51–59
1945.0	109–126	968.5	68–76
1739.9	127–142	1309.7	77–87
2134.9	150–169	1276.3	88–98
1501.6	170–183	2729.4	226–251
1817.5	191–207	834.4	252–295
		2081.2	259–277
		1676.8	278–291
		1188.5	296–304
		1806.9	305–320
		837.5	330–337
		1285.7	348–358
		1103.7	364–373
		2543.2	374–395
		1872.9	396–412
		2743.3	420–442
		787.5	443–450

**Table 4:** Peptides identified after digestion of Bevacizumab with trypsin by LC/MS/MS analysis.

Light Chain		Heavy Chain	
Measured Molecular Weight (Da)	Peptide Length	Measured Molecular Weight (Da)	Peptide Length
1877.9	1–18	1881.1	1–19
2743.3	19–42	2139.9	20–38
1761.9	46–61	2517.3	44–65
4602.4	62–103	1044.5	68–76
1945.0	109–126	1282.6	77–87
1739.9	127–142	1232.7	88–98
2134.9	150–169	3322.6	99–127
1501.6	170–183	1185.7	128–139
1817.5	191–207	1263.7	140–153
		2729.4	229–254
		834.4	255–261
		2081.2	262–280
		1676.8	281–294
		1188.5	299–307
		1806.9	308–323
		837.5	333–340
		1285.7	351–361
		1103.7	367–376
		2543.2	377–389
		1872.9	399–415
		2743.3	423–445

**Table 5:** Peptides identified after digestion of Cetuximab with trypsin by LC/MS/MS analysis.

Light Chain		Heavy Chain	
Measured Molecular Weight (Da)	Peptide Length	Measured Molecular Weight (Da)	Peptide Length
1923.2	1–18	3504.8	6–38
1787.9	25–39	2569.3	44–66
1265.4	50–61	754.4	76–81
4507.9	62–103	1848.8	82–97
1945.0	109–126	2904.4	98–123
1739.9	127–142	1185.6	124–135
2134.9	150–169	1263.6	136–149
1501.6	170–183	2729.4	229–254
1817.5	191–207	834.4	255–261
		2081.2	262–280
		1676.8	281–294
		1188.5	299–307
		1806.9	308–323
		837.5	333–340
		1285.7	351–361
		1103.7	367–376
		2543.2	377–389
		1872.9	399–415
		2743.3	423–445
		787.5	446–453

**Table 6:** Stability data expressed as average MW of Trastuzumab, Bevacizumab, and Cetuximab after LC-MS/MS analysis.

	Trastuzumab (Mw ± st. dev.)	Bevacizumab (Mw ± st. dev.)	Cetuximab (Mw ± st. dev.)
Day 1	145,534 ± 4	149,195 ± 4	145,783 ± 5
Day 2	145,533 ± 5	149,195 ± 4	145,786 ± 3
Day 3	145,534 ± 2	149,198 ± 3	145,784 ± 4
Day 4	145,536 ± 5	149,196 ± 5	145,784 ± 5
Day 5	145,532 ± 2	149,197 ± 3	145,784 ± 4
Day 8	145,535 ± 3	149,194 ± 6	145,787 ± 2
Day 10	145,538 ± 6	149,199 ± 8	145,783 ± 6
Day 12	145,530 ± 5	149,197 ± 2	145,782 ± 5
Day 15	145,534 ± 5	149,195 ± 3	145,782 ± 6
Day 18	145,535 ± 2	149,195 ± 7	145,784 ± 4
Day 22	145,531 ± 2	149,197 ± 4	145,783 ± 3

## Data Availability

The original contributions presented in this study are included in the article. Further inquiries can be directed at the corresponding author.

## Abbreviations

API	Active Pharmaceutical Ingredient
ABR	Abraxane^®^
ALI	Alimta^®^
ASL Na3	Local Health Authority Naples 3 South
AVA	Avastin^®^
ERBI	Erbitux^®^
GERPAC	Evaluation and Research Group on Protection in Controlled Atmosphere
GHPMP	Good Hospital Pharmacy Manufacturing Practice
HER	Herceptin^®^
JEV	Jevtana^®^
MS	Mass spectroscopy
PBS	Phosphate-buffered saline
PDI	Polydispersity index
SDS_PAGE	Sodium Dodecyl Sulfate—PolyAcrylamide Gel Electrophoresis
SEC	Size exclusion chromatography
SFPC	French Society of Clinical Pharmacy
SmPCs	Summary of product characteristics
UFA	Unità Farmaci Antiblastici-Centralized Antiblastic Drug Unit
USP	United States Pharmacopeia

## References

[1] Mathur S., Sutton J. (2017). Personalized medicine could transform healthcare (Review). Biomed. Rep..

[2] Chan I.S., Ginsburg G.S. (2011). Personalized medicine: Progress and promise. Annu. Rev. Genom. Hum. Genet..

[3] Savchenko E., Bunimovich-Mendrazitsky S. (2024). Investigation toward the economic feasibility of personalized medicine for healthcare service providers: The case of bladder cancer. Front. Med..

[4] Khashayar V., Jason R., Linda S.F., Samir F. (2007). Optimizing physician staffing and resource allocation: Sine-wave variation in hourly trauma admission. J. Trauma.

[5] Arnedos M., Soria J.-C., Andre F., Tursz T. (2014). Personalized treatments of cancer patients: A reality in daily practice, a costly dream or a shared vision of the future from the oncology community?. Cancer Treat. Rev..

[6] Sewell G., Plumridge R. (2001). Dose-banding of cytotoxic drugs: A new concept in cancer chemotherapy. Am. J. Health-Syst. Pharm..

[7] Gonzalez-Angulo A.M., Hennessy B.T., Mills G.B. (2010). Future of personalized medicine in oncology: A systems biology approach. J. Clin. Oncol..

[8] Buma A.I., Piet B., Ter Heine R., van den Heuvel M.M. (2023). Integrating treatment cost reduction strategies and biomarker research to reduce costs and personalize expensive treatments: An example of a self-funding trial in non-small cell lung cancer. Front. Pharmacol..

[9] Massimiliano B., Raffaele Di F. (2018). Editorial: The Real Impact of Target Therapy in Cancer Patients: Between Hope and Reality. Curr. Cancer Drug Targets.

[10] Handorf E.A., McElligott S., Vachani A., Langer C.J., Bristol Demeter M., Armstrong K., Asch D.A. (2012). Cost effectiveness of personalized therapy for first-line treatment of stage IV and recurrent incurable adenocarcinoma of the lung. J. Oncol. Pract..

[11] Bakshi M., Singh S. (2002). Development of validated stability-indicating assay methods—Critical review. J. Pharm. Biomed. Anal..

[12] Adade C.A., Benabbes M., Belahcen M.J., Rahali Y. (2020). Centralization impact and cost-saving study in a Moroccan hospital’s centralized unit of chemotherapy preparation. J. Oncol. Pharm. Pract..

[13] Bouwman Y., Andersen L.M. (2012). GMP and preparation in hospital pharmacies. Eur. J. Hosp. Pharm. Sci. Pract..

[14] Bardin C., Astier A., Vulto A., Sewell G., Vigneron J., Trittler R., Daouphars M., Paul M., Trojniak M., Pinguet F. (2011). Guidelines for the practical stability studies of anticancer drugs: A European consensus conference. Ann. Pharm. Françaises.

[15] International Conference of Harmonization (ICH) Guidelines for Stability. https://www.ich.org/page/quality-guidelines.

[16] Rai K., Potphode P., Gupta C., Rao N. (2020). Comparitive study between ICH guideline and anvisa guideline. Int. J. Res. Pharm. Chem..

[17] Astier A. (2012). Practical stability studies: A powerful approach for reducing the cost of monoclonal antibodies. Eur. J. Oncol. Pharm..

[18] Chiumente M., Rivano M., Severino D., Bertoli S., Gasbarro A.R., Santeramo R., Pasqualini A., Provasi R., Mengato D., Palozzo A.C. (2023). Oncostability: Stability of reconstituted and diluted anticancer medicines for a possible extension of use. Eur. J. Hosp. Pharm..

[19] Fasola G., Aita M., Marini L., Follador A., Tosolini M., Mattioni L., Mansutti M., Piga A., Brusaferro S., Aprile G. (2008). Drug waste minimisation and cost-containment in Medical Oncology: Two-year results of a feasibility study. BMC Health Serv. Res..

[20] Ioele G., Chieffallo M., Occhiuzzi M.A., De Luca M., Garofalo A., Ragno G., Grande F. (2022). Anticancer Drugs: Recent Strategies to Improve Stability Profile, Pharmacokinetic and Pharmacodynamic Properties. Molecules.

[21] Vigneron J., Astier A., Trittler R., Hecq J.D., Daouphars M., Larsson I., Pourroy B., Pinguet F. (2013). SFPO and ESOP recommendations for the practical stability of anticancer drugs: An update. Ann. Pharm. Françaises.

[22] EMA Summary of Product Characteristics (Alimta). https://www.ema.europa.eu/en/medicines/human/EPAR/alimta.

[23] EMA Summary of Product Characteristics (Abraxane). https://www.ema.europa.eu/en/medicines/human/EPAR/abraxane.

[24] EMA, Summary of Product Characteristics (Jevtana). https://www.ema.europa.eu/en/medicines/human/EPAR/jevtana.

[25] EMA Summary of Product Characteristics (Herceptin). https://www.ema.europa.eu/en/medicines/human/EPAR/herceptin.

[26] EMA Summary of Product Characteristics (Avastin). https://www.ema.europa.eu/en/medicines/human/EPAR/avastin.

[27] EMA Summary of Product Characteristics (Erbitux). https://www.ema.europa.eu/en/medicines/human/EPAR/erbitux.

[28] Deeb T., Brunotte R., Hubenia O., Leal-Marin S., Glasmacher B. (2024). Advancements in Cryogenic Freezing Instrumentation for Cryopreservation. Annu. Rev. Heat Transf..

[29] de Lemos M.L., Hamata L. (2007). Stability issues of parenteral chemotherapy drugs. J. Oncol. Pharm. Pract..

[30] SFPC (French Society of Clinical Pharmacy), GERPAC (Evaluation and Research Group on Protection in Controlled Atmosphere) (2013). Methodological Guidelines for Stability Studies of Hospital Pharmaceutical Preparations.

[31] (2021). United States Pharmacopeia 44, National Formulary 39.

[32] Venkatesh B., Mathrusri Annapurna M., Pramadvara K. (2015). Analytical stress degradation studies of cabazitaxel (a semi synthetic natural taxoid) using liquid chromatography. Pharm. Methods.

[33] D’Huart E., Sacrez M., Vigneron J., Sobalak N., Demoré B. (2023). Physicochemical stability of Cabazitaxel Zentiva^®^ solution in vials after opening and diluted solutions in three infusion bags. Pharm. Technol. Hosp. Pharm..

[34] Manning M.C., Chou D.K., Murphy B.M., Payne R.W., Katayama D.S. (2010). Stability of Protein Pharmaceuticals: An Update. Pharm. Res..

[35] Hawe A., Wiggenhorn M., van de Weert M., Garbe J.H.O., Mahler H.-c., Jiskoot W. (2012). Forced Degradation of Therapeutic Proteins. J. Pharm. Sci..

[36] Kaur H. (2021). Stability testing in monoclonal antibodies. Crit. Rev. Biotechnol..

[37] Vieillard V., Le Guyader G., Jallades A., Astier A. (2023). Extended physicochemical stability of cetuximab in opened vials and infusion bags when stored at 4 °C and 25 °C. J. Oncol. Pharm. Pract..

[38] Carpanese D., Rossi V., Di Paolo V., Quintieri L., Penna A., Zuccolotto G., Sebellin J., Saran C., Pipitone F., Miolo G. (2024). Prolonging the stability of cetuximab (Erbitux^®^) and panitumumab (Vectibix^®^): An orthogonal assessment of physicochemical, biological and microbiological properties of original opened glass vials and diluted saline preparations. Int. J. Pharm..

[39] Aebersold R., Mann M. (2016). Mass-spectrometric exploration of proteome structure and function. Nature.

[40] Izzo V., Charlier B., Bloise E., Pingeon M., Romano M., Finelli A., Vietri A., Conti V., Manzo V., Alfieri M. (2018). A UHPLC–MS/MS-based method for the simultaneous monitoring of eight antiblastic drugs in plasma and urine of exposed healthcare workers. J. Pharm. Biomed. Anal..

[41] Charlier B., Coglianese A., De Rosa F., Cozzolino A., Boccia G., Borrelli A., Capunzo M., Genovese G., De Caro F., Filippelli A. (2023). A LC-MS/MS based methodology for the environmental monitoring of healthcare settings contaminated with antineoplastic agents. J. Public Health Res..

[42] Nalenz H., Köpf E., Dietel E. (2018). Prolonged In-use Stability of Reconstituted Herceptin in Commercial Intravenous Bags. Int. J. Pharm. Compd..

